# Bordeaux Method of Dressing Wounds

**Published:** 1877-11

**Authors:** 


CICATRIZATION OF LARGE WOUNDS UNDER THE SO-CALLED BORDEAUX DRESSING.—Dr. Azam has collated the statistics of a large number of cases of extensive wounds, especially amputations, which were dressed in the manner peculiar to Bordeaux. In this dressing the aim is to secure immediate union of the entire surface of the wound. Its three cardinal points are drainage, deep sutures, and superficial sutures. After the hemorrhage has been controlled as thoroughly as is possible, a thick drainage tube is laid in the deepest part of the wound--in amputations, behind the bone—and fastened over the limb between the two angles of the wound. The flaps are next united at their bases by deeply placed double sutures of very fine silver wire, and the ends of the sutures are twisted over a piece of a sound. These sutures are from two to three in number, according to the thickness of the flaps, and are situated from four to five centimetres from the edges of the flaps. Finally the wound is carefully closed by harelip pins and twisted sutures, supported by strips of charpie dipped in collodion. The entire part is then enveloped in wadding, except at the places where the drainage tubes lie, which are covered with charpie to soak up the discharge. On the second or third day the harlip pins are removed and the deep sutures loosened. The dressing is renewed every three or four days. Cicatrization is completed in from ten to twenty days.

Dr. Azam saw an amputation of the thigh heal up in ten days, and an amputation of the leg in eleven days. In 202 amputations, of which 63 were amputations of the leg or thigh, there were twelve deaths; out of 30 amputations of the thigh there were five deaths, and out of 33 amputations of the leg there were three.—*Allg. Med. Cent. Zeit.*, Aug. 8th.

